# Facile One-Step Synthesis of PVDF Bead-on-String Fibers by Pressurized Gyration for Reusable Face Masks

**DOI:** 10.3390/polym14214498

**Published:** 2022-10-24

**Authors:** Ruiran Huang, Yanqi Dai, Jubair Ahmed, Mohan Edirisinghe

**Affiliations:** Department of Mechanical Engineering, University College London, Torrington Place, London WC1E 7JE, UK

**Keywords:** pressurized gyration, polyvinylidene fluoride (PVDF), bead-on-string fiber, fiber morphology, β-phase, hydrophobicity

## Abstract

Single-use face masks pose a threat to the environment and are not cost-effective, which prompts the need for developing reusable masks. In this study, pressurized gyration (PG) successfully produced bead-on-string polyvinylidene fluoride (PVDF) fibers with fiber diameters ranging from 2.3 μm to 26.1 μm, and bead diameters ranging from 60.9 μm to 88.5 μm by changing the solution parameters. The effect of the solution parameters on the crystalline phase was studied by Fourier-transform infrared spectroscopy (FT-IR), where the β-phase contents of PG PVDF fibers reached over 75%. The fiber morphology and β-phase contents of PG PVDF fibers indicated the potential mechanical and electrostatic filtration efficiency of PG PVDF fibers, respectively. Additionally, the hydrophobicity was investigated by static water contact angle tests, and the PVDF fibers showed superior hydrophobicity properties (all samples above 125°) over commercial polypropylene (PP) single-use masks (approximately 107°). This study supports the notion that the PG PVDF fiber mats are a promising candidate for future reusable face masks.

## 1. Introduction

The COVID-19 pandemic has demonstrated the importance of developing personal protective equipment (PPE), especially air filtration devices, such as face coverings. Most current face masks are made of melt-blown polypropylene (PP) non-woven fabrics with large fiber diameters that are insufficient at filtering aerosols efficiently [1]. Even though electrostatic treatment can improve the filtration efficiency of these fabrics, the electrostatic charges can wane after several hours [2]. Thus, these masks are disposable, creating massive polymer waste and polluting the environment. In addition, single-use PP masks are not a cost-effective product. According to a recent investigation, single-use masks are three times more expensive than reusable masks in their usage life, from manufacture to disposal [3]. Therefore, the need to develop reusable face masks with improved filtration efficiency has become urgent.

Both the mechanical and the electrostatic filtration efficiency of filters can be altered by changing the fiber morphology to improve filtration efficiency. An example is reducing the fiber diameter by changing the solution parameters in electrospinning. Fibers with smaller diameters have better mechanical capture properties by diffusion and interception, and improved electrostatic capture by storing more charge during corona discharge [4]. Another example is the bead-on-string structure of certain fibers, which can act as physical separators between fibers, thus optimizing the fiber packing density [5]. The cavity structure caused by the beads can sufficiently reduce the pressure drop and improve breathability by providing a pathway for airflow [6]. Furthermore, beads can also increase the impact area of filters with particle matter (PM), thereby strengthening the co-effects of interception, diffusion, and inertance to capture particles [7]. Conventionally, bead-on-string fibers for filtration are produced by electrospinning, owing to the easily adjustable processing parameters (e.g., viscosity and conductivity of solutions) [8]. Nonetheless, the high voltage required for electrospinning restricts its ability to scale up the production of fibrous filters industrially.

Pressurized gyration (PG) is a technique developed in 2013 which utilizes centrifugal force and infused gas pressure to facilitate fiber production [9]. During the PG process, polymer jets are ejected from the orifices of a vessel rotating at high speed and then stretched by centrifugal force. Meanwhile, gas is blown into the vessel to facilitate solvent evaporation and jet elongation, thus forming finer fibers. Like electrospinning, PG also possesses easily adjustable processing parameters (e.g., rotating speed, working pressure), which enables the fabrication of fibers with versatile morphologies. In addition, PG fiber diameters can be controlled by changing the solution parameters (e.g., polymer concentrations, molecular weight), which affect the viscosity force and the surface tension during PG [10,11,12]. PG beaded fibers with different bead density, bead sizes, and inter-bead spaces can also be manufactured by altering the working parameters (pressure and rotation speed) and solution concentrations [13].

Recently, polyvinylidene fluoride (PVDF) nanofibers have garnered much attention in the application of air filtration. PVDF possesses low surface energy, thus having a relatively large water contact angle of approximately 93.4° [14]. Due to its excellent hydrophobic properties, superhydrophobic PVDF membranes can be obtained by simply increasing the surface roughness according to Cassie’s theory [15]. In addition, there are five kinds of polymorphic structures of PVDF in the form of α- and δ-(TGTG’), β-(TTT), γ, and ε-(T3GT3G′) (T for trans and G for gauche) [16]. In particular, PVDF with a high content of β-phase possesses excellent piezoelectric properties, which makes it a favorable material for piezoelectric nanogenerators (PENG) [17]. PVDF nanofiber-based nanogenerators have the potential to be manufactured into self-charged face masks, which can generate electrical charges by the motion of the human body, thus improving the electrostatic filtration efficiency [17,18,19]. To increase the β-phase content in PVDF, chemical, thermal or physical treatments can been applied, such as the dissolution of PVDF in different solvents, electrical poling, and mechanical stretching [20]. Electrospinning has been regarded as the most common method to generate PVDF fibers with a high fraction of β-phase since the high-field electrical poling in electrospinning can induce the formation of β-phase and re-orient the alignment of -CH_2_-CF_2_ dipoles in the PVDF [21]. However, electrospinning requires a high-voltage electrical field, and the production rate is typically slow. Thus, other fiber processing techniques have been investigated to overcome this limitation. As reported by Ibtehaj et al., fast-centrifugal spinning can produce a high degree of β-chain alignment in PVDF fibers since the stretching effect induced in centrifugal spinning can elongate and unfold the spherulite α-phase into the all-trans planar zigzag β-phase [22]. In another research study, the authors used a binary solvent system of DMF and acetone to promote the α- to β-phase transformation via dipolar intermolecular interaction and the centrifugal force to orientate -CH_2_-CF_2_ dipoles in PVDF parallel to the drawing direction [20]. Additionally, solution blow spinning was investigated because the polymer jet’s tension caused by air pressure from the spinning nozzle is also a unidirectional mechanical stretching that can produce a high fraction of the β-phase [23].

PG is a technique combining solution blow spinning and centrifugal spinning. Thus, it is hypothesized that PG can also be used to process PVDF fibers with desirable morphologies and piezoelectric properties that can be a promising candidate for reusable face masks. Compared with blow spinning, PG avoids the risk of needle blockage [23]. It also exhibits great potential in mass production considering that there are 24 orifices in PG, while only one fiber emerging from a single needle in blow spinning. Therefore, PG can produce more fibers. The introduction of gas into the centrifugal spinning vessels enables faster solvent evaporation, thus its fiber production rate is much higher than that of centrifugal spinning [9]. The gas in PG also contributes to the formation of beads and porous structures in the fibers, which is beneficial for filtration efficiency. In this work, we specifically showcase the feasibility in the production of PVDF hydrophobic fibrous mats using PG. By varying polymer concentration and the ratio of the binary solvent, PG PVDF fiber mats with different surface morphologies and contents of β-phase were obtained. We can achieve beneficial surface characteristics such as nanopores and bead-on-string fiber morphology, which increase the available surface area for mechanical filtration. Here, we show that a very high production rate can be achieved by using pressurized gyration, which has the potential to meet soaring demands of the face-mask industry.

## 2. Materials and Methods

### 2.1. Materials

Polyvinylidene fluoride (PVDF, Mw ≈ 275,000 g mol^−1^, CAS: 24937-79-9), N, N-dimethylformamide (DMF, CAS: 68-12-2), and acetone (CAS: 67-64-1) were purchased from Sigma–Aldrich (Dorset, UK). All chemicals were of analytical grade and were used as received.

### 2.2. Preparation and Characterization of PVDF SOLUTIONS

PVDF solutions with different polymer concentrations (15%, 20%, 25% (*w*/*v*)) were obtained by dissolving PVDF pellets in DMF and acetone at different ratios (5/5, 4/6, 3/7 (*v*/*v*)). A magnetic stirrer was used to accelerate the dissolution process at a hotplate temperature 60 ℃ for 24 h to obtain homogeneous solutions. Samples were labeled as in Table 1.

The viscosity of the PVDF solutions was characterized by a Brookfield DV-III Ultra Viscometer by using a small-sample adaptor attached to a SCV-18 spindle (50 A643, Fisher Scientific, Waltham, MA, USA). The surface tension was characterized by a Krüss digital tensiometer K9 using the Du Nouy ring method (Surface Tensiomat model 21, Fisher Scientific, Waltham, MA, USA). All equipment was calibrated before beginning experiments, and the tests were carried out three times for each sample at ambient temperature (20–25 °C).

### 2.3. Pressurized Gyration

**Figure 1:**
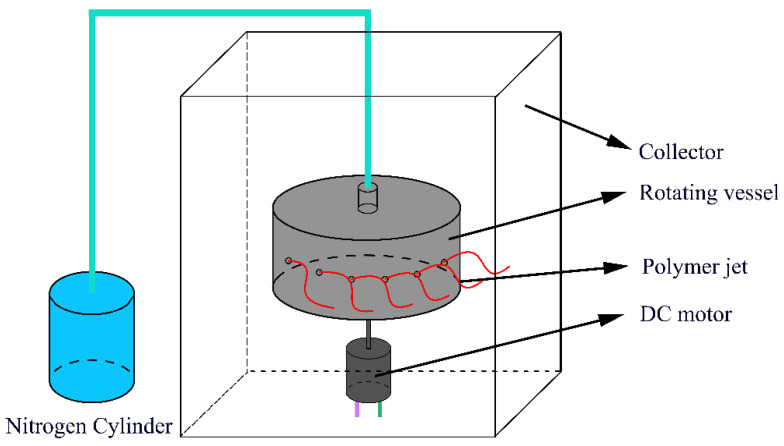
The pressurized gyration setup, showing the rotating vessel and the nitrogen gas pressure infusion.

A pressurized gyration apparatus was used to spin the solutions mentioned above (Figure 1). Specifically, 3.5 mL of PVDF solution was injected into the rotating aluminum cylinder vessel (58 mm inner diameter, 60 mm outer diameter, 35 mm height) which has 24 orifices (0.5 mm diameter) on the wall of the vessel. The bottom of the vessel was connected to a DC motor which provided a rotating speed of 13,000 rpm, and the top of the vessel was connected to a constant nitrogen gas stream (N_2_) which provided a flow pressure of 0.2 MPa. In order to collect PVDF fibers, the PG apparatus was placed in a cabinet, where the collecting distance, the shortest vertical distance between the orifices and the wall of the collector from the front facing view of the gyration vessel, was kept at 100 mm. Fibers were obtained at ambient conditions (temperature of 20–25 °C and relative humidity of 45–55%). All experiments were repeated three times to calculate the production rate and production yield. The yield and the production rate were calculated using the following Equations (1) and (2), respectively. Additionally, samples with high production rates were selected for further experiments.

(1) $$\eta =\frac{w_{f}}{w_{0}}$$

(2) $$P_{r}=\frac{w_{f}}{t_{s}}$$

η is defined as the yield. W_0_ is the mass of input polymer pellets, and W_f_ is the mass of produced fibers. P_r_ is defined as the production rate. t_s_ is the time required to produce a certain quantity of fibers.

### 2.4. Characterization

#### 2.4.1. Scanning Electron Microscopy (SEM)

Fiber morphology was characterized by SEM (EVO LS 10, ZEISS SEM Instrument, Jena, Germany). All micrographs were taken at an acceleration voltage of 20 kV. Adobe^®^ Lightroom CC (2015.5, San Jose, CA, USA) was used to improve the contrast and brightness of the images. ImageJ (v1.51, Bethesda, MD, USA) was used to measure the size of the beads as well as the fiber diameters, after which the diameter frequency distributions of the fibers and beads were obtained via Origin Pro (2021, Northampton, MA, USA). The mean values and the standard deviation of fiber and bead diameters were also calculated. The mean values indicate the average diameters of fibers and beads, while the standard deviations indicate their diameter distribution. The measurements were repeated on at least 100 fibers/beads randomly picked. N in the distribution figures represented the number of fibers or beads measured.

#### 2.4.2. Attenuated Total Reflection Fourier Transform Infrared Spectroscopy (ATR-FTIR)

Characterization of the β-phase in PVDF fibers was conducted using ATR-FTIR. (Alpha FTIR Spectrophotometer, Bruker, Bremen, Germany) The transmission spectra was recorded between 400–4000 cm^−1^ wavelength with a resolution of 4 cm^−1^ and 16 scans for each sample at ambient temperature (25 °C). The background spectrum was measured before testing the samples. The measurement of each sample was repeated three times to obtain the standard deviation of the β-phase fraction of the PVDF fibers. The resulting were normalized with 1072 cm^−1^ and plotted using Origin Pro (software).

The relative β-phase fraction of PVDF fibers was calculated using the following equation based on the FTIR spectra [24].

(3) $$F\left(\beta \right)=\frac{A_{\beta }}{\frac{K_{\beta }}{K_{\alpha }}A_{\alpha }+A_{\beta }}=\frac{A_{\beta }}{1.26A_{\alpha }+A_{\beta }}$$

*A*_α_ and *A*_β_ are the absorbance intensities of α-phases at 763 cm^−1^ and β-phases at 840 cm^−1^, respectively. Moreover, *K*_α_ and *K*_β_ are the α and β-phases absorbance coefficients with values of 6.1 × 10^4^ cm^2^ mol^−1^ and 7.7 × 10^4^ cm^2^ mol^−1^, respectively.

#### 2.4.3. Static Surface Hydrophobicity Measurement

PVDF fibers mats were prepared into a thickness of 1 mm and were adhered to glass sample plates by using carbon tape. Following this, the water contact angles were measured by the sessile drop method. The equipment syringe was placed vertically and filled with distilled water. A water droplet of approximately 35 μL was dropped onto each of the PVDF fibers mats. The surface baselines were assigned manually, followed by the measurement of water contact angles via an optical tensiometer (Theta Attention, Biolin Scientific, Espoo, Finland).

## 3. Results and Discussion

### 3.1. Influence of the PG Solution Parameters on Fiber Morphology

**Figure 2:**
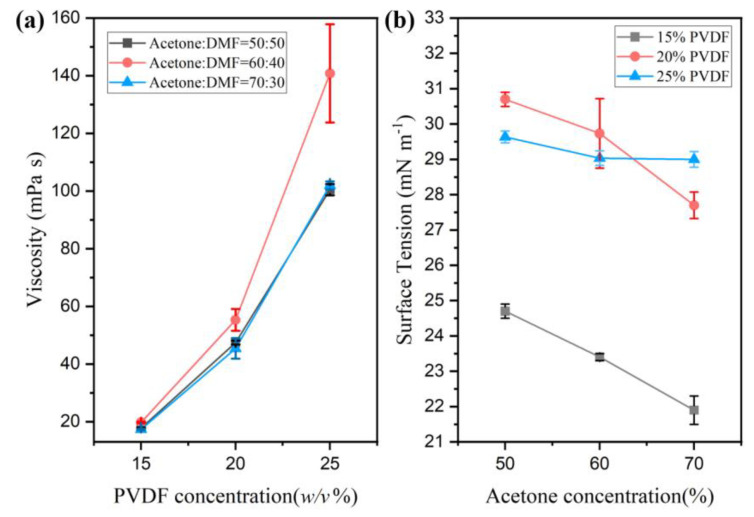
PVDF solution properties: (**a**) viscosity at different polymer concentrations; (**b**) surface tension at different acetone concentrations.

Fiber formation is closely dependent on the viscosity and surface tension of the polymer solutions. As shown in Figure 2, polymer concentration was the dominant factor influencing viscosity, while the solvent ratio played a prominent role in controlling the surface tension. Specifically, the solution viscosity increased as the polymer concentration increased. Since there is insufficient chain entanglement under low viscosity, the fibers can only be formed when the polymer concentration reaches a certain critical value [25]. It should be noted that the viscosity of the PVDF solution with 60% acetone for all tested polymer concentrations is higher than the other two groups, especially when the polymer concentration is higher. On one hand, the viscosity of DMF is higher than that of acetone and has a closer solubility parameter suitability with PVDF compared to acetone. This can contribute to the higher viscosity of the PVDF solution using 60% acetone [23]. However, when the DMF ratio is increased to 50% in the solution, the high polarity of DMF contributes to the orientation of the polymer configuration [26]. This may lead to variances in the degrees of chain entanglement, leading to the lower viscosity of the PVDF solution using 50% acetone. If the surface tension of the solution is too high, the centrifugal force and the gas pressure cannot overcome the surface tension [9,27,28]. Thus, polymer jets cannot be formed. In this research, all PVDF solutions maintained a compatible surface tension that allowed fiber production. Additionally, a downward trend of surface tension can be observed when the acetone concentration increased because the surface tension of acetone is lower than that of DMF.

#### 3.1.1. Effects of Polymer Concentrations on Fiber Morphology

**Figure 3:**
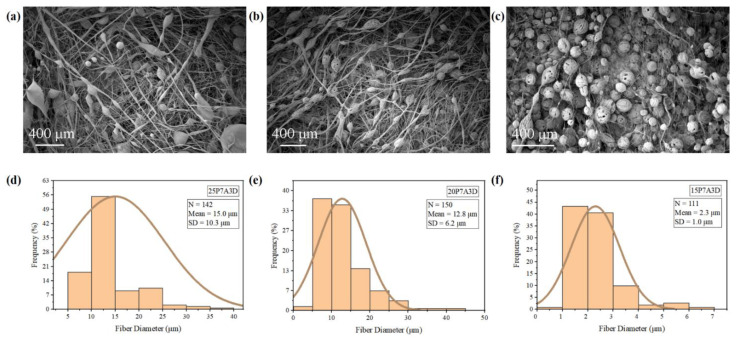
SEM images and fiber diameter distributions of PVDF fibers: (**a**,**d**) 25P7A3D; (**b**,**e**) 20P7A3D; (**c**,**f**) 15P7A3D.

Lower bead density and elongated beads can be observed in the fibers processed using higher polymer concentrations (Figure 3a,b), while there were more circular beads in the 15P7A3D (Figure 3c). As reported by Hong and Edirisinghe et al., a greater amount of chain entanglement can stabilize the polymer flow [13]. Thus, the polymer jets can be elongated and dried as long fibers [13]. In contrast, the solutions with lower polymer concentrations are easily disturbed by the airflow and are more likely to deposit on the collector as droplets. Figure 3d–f shows that the average fiber diameter of PVDF decreased as the polymer concentrations of the spinning solutions decreased. This phenomenon can be attributed to the higher viscosity and slower solvent evaporation rate of the PVDF solutions at higher polymer concentrations. Consequently, PVDF with a larger fiber diameter was produced at higher polymer concentrations.

**Figure 4:**
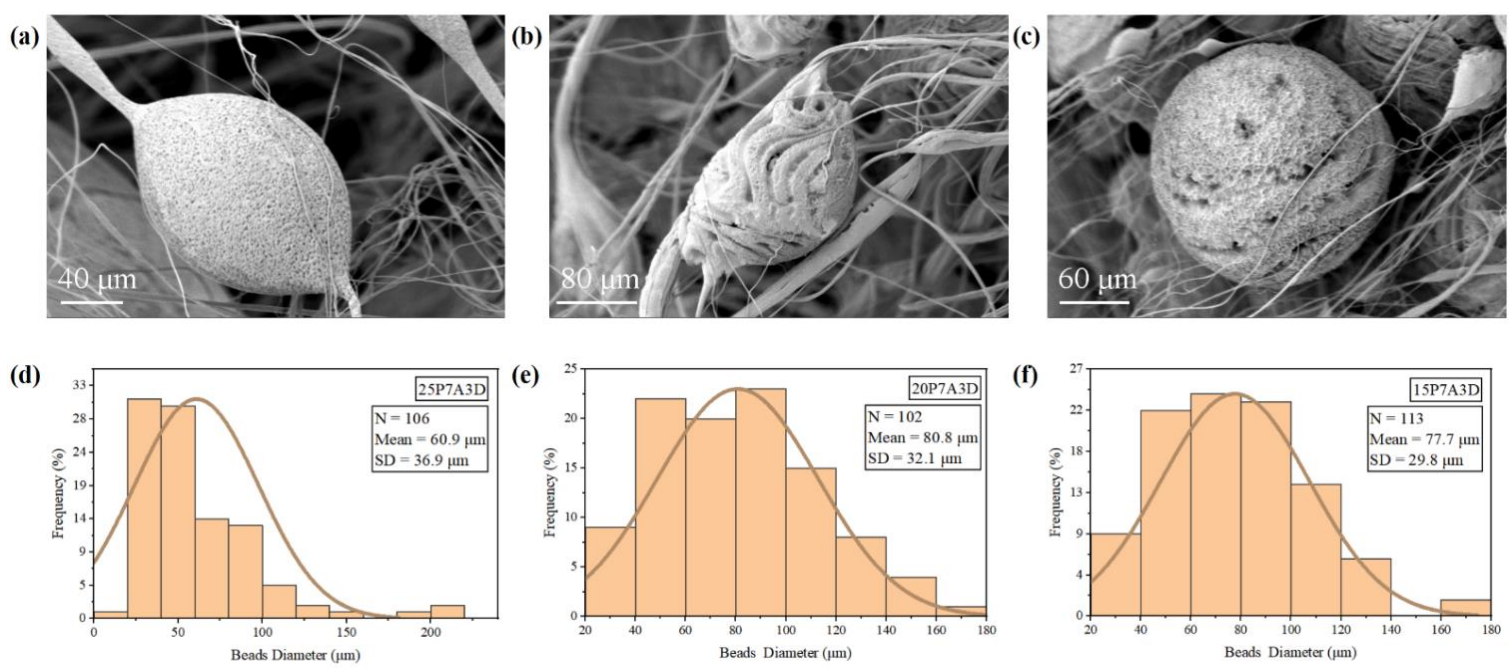
SEM images of beads on the PVDF fibers and bead diameter distributions: (**a**,**d**) 25P7A3D; (**b**,**e**) 20P7A3D; (**c**,**f**) 15P7A3D.

Beads from all the samples exhibited rough surfaces (Figure 4a–c). The nanopores found on the surface of the beads can be attributed to phase separation and evaporation of the solvent during PG. The gas facilitated the solvent evaporation, which induced temperature drop in fibers, leading to the condensation of water vapor [10]. After these water droplets evaporated, the pores were formed in the place where they resided [29]. It seems that the beads of the 20P7A3D sample consisted of retractable fibers (Figure 4b), stemming from the instability flow during the fiber stretching and collecting process. In addition, a large bead diameter distribution can also be observed from all samples because instability and elastic recoil usually occurs between beads and fibers [13]. This phenomenon may result from the balance between inertia, surface tension, and viscoelasticity of the polymer solution. [13].

#### 3.1.2. Effects of Solvent Ratio on Fiber Morphology

**Figure 5:**
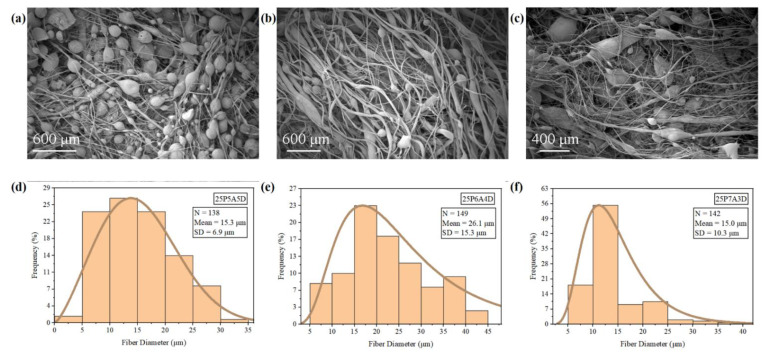
SEM images and fiber diameter distributions of PVDF fibers: (**a**,**d**) 25P5A5D; (**b**,**e**) 25P6A4D; (**c**,**f**) 25P7A3D.

Based on Figure 5, the fiber diameter of the 25P7A3D sample and the 25P5A5D sample was smaller than that of the 25P6A4D sample, and there were more beads present in these two groups. This resulted from the low viscosity of the 25P7A3D and 25P5A5D samples which caused instability of the PVDF jets. Since there were fewer chain entanglements, the PVDF jet could be easily cut off by high air pressure, forming beads [23]. From the perspective of surface tension, at a high DMF concentration (25P5A5D), the surface tension of the PVDF solution was higher, and there was a low ratio between the viscous force and surface tension. This led to the breaking of the polymer jet, forming a larger number of beads [30]. However, as the acetone ratio increased to 70%, additional beads were observed in the 25P7A3D sample compared to that of the 25P6A4D sample. In this scenario, evaporation of the solvent was too rapid due to the high concentration of acetone. Thus, polymer extension was impeded, and beads were formed.

**Figure 6:**
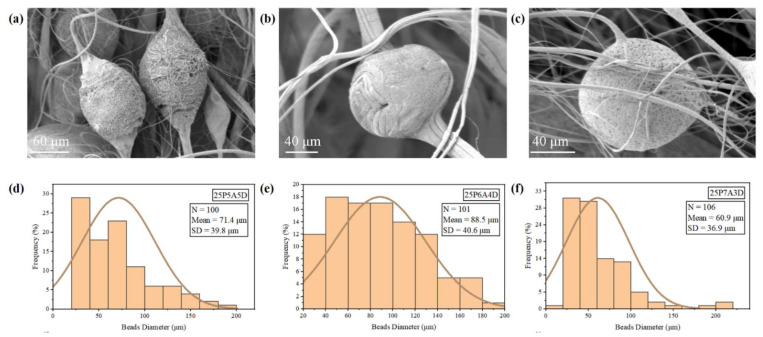
SEM images of beads on PVDF fibers and bead diameter distributions: (**a**,**d**) 25P5A5D; (**b**,**e**) 25P6A4D; (**c**,**f**) 25P7A3D.

From looking at Figure 6, the surface roughness is seen to have increased when the solvent ratio was closer to 1. In the 25P5A5D group, larger pores can be easily observed on the PVDF beads (Figure 6a). These pores can be associated with the use of the binary solvent system. Acetone and DMF have very different volatilities, resulting from the difference in boiling points. Thus, they vaporized at different instances during the PG process, promoting the formation of pores [12]. In addition, DMF has good miscibility with PVDF, while acetone is a non-solvent of PVDF. Thus, phase separation occurred during the fiber formation process, and the nucleation and growth of the polymer during phase separation gave rise to pores on the beaded fibers. As the acetone concentration increased to 60%, irregular patterns could be observed on the beads (Figure 6b). This may be attributed to the intertwining of fibers during the condensation of unstable polymer flow.

In summary, there is a relationship between the bead density and the fiber diameter of the PG PVDF fibers. Namely, groups with thinner fiber diameters usually presented a higher bead density. Low viscosity can lead to faster evaporation of the solvent and a lower flow rate, contributing to thinner fiber diameters. It can also result from the instability of the polymer flow, which will eventually contribute to the formation of beads. Therefore, it can be concluded that by altering the solution parameters to adjust viscosity, surface tension and solvent evaporation rate, PVDF fibers with a high bead density and small fiber diameters can be obtained. However, it should be noted that even though thinner fibers and beads can play a vital role in improving the mechanical filtration efficiency, the beads and thin fiber diameters will result in poorer mechanical properties [4]. Thus, there will be a trade-off between the fiber diameters, beads, and the mechanical strength of the fiber mats. In addition, the use of a binary solvent systems increased the surface roughness of all the tested groups, which supports filtration efficiency. It is assumed that nanopores on the beads in the 25P5A5D sample will be conducive to the absorption of particles as reported by Wang et al., while the microscale rough surface of beads in the 25P6A4D sample can also provide a larger specific surface area for the absorption of particles [5].

### 3.2. β-Phase Formation of PG PVDF

**Figure 7:**
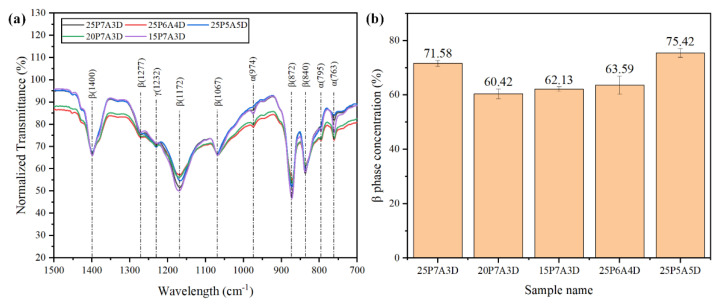
(**a**) FTIR spectra of the PVDF fibers processed by different polymer concentrations and solvent ratios; (**b**) the β-phase fraction of PVDF processed by different polymer concentrations and solvent ratios.

As shown in Figure 7a, the typical peaks related to the β-phase can be clearly observed at 1400 cm^−1^, 1172 cm^−1^, 1067 cm^−1^, 872 cm^−1^, and 840 cm^−1^ [20,22]. These indicate the formation of the β-phase in PG PVDF fibers processed by all polymer concentrations and solvent ratios. The mechanism behind the β-phase formation may be mechanical stretching resulting from the centrifugal force and air pressure, which can elongate and unfold the spherulite α-phase into the all-trans planar zigzag β-phase [20,23].

Figure 7b shows that the relative β-phase fraction of the 25P7A3D sample was much higher than that of the samples prepared with lower polymer concentrations, while PVDF fibers of lower polymer concentration show similar β-phase fraction. This phenomenon occurs from the much higher viscosity of the 25P7A3D sample compared to the other two groups. At 25% (*w*/*v*) polymer concentration, there was significantly lower solvent evaporation rate and a greater polymer flow ejecting from the orifices at a fixed size. This led to more extensive mechanical stretching in the PVDF fibers. Accordingly, there was an improvement to the relative β content in the PVDF fibers. Generally, the β-phase fraction increased as the concentrations of DMF increased. This can be attributed to two main reasons; firstly, the intermolecular interactions between the high polarity DMF molecules and -CH_2_-CF_2_ dipoles of PVDF were conducive to β-phase formation [22]. Secondly, decreasing concentrations of acetone resulted in a lower solvent evaporation rate, which indicated more mechanical stretching, thus a higher fraction of the β-phase. However, the 25P7A3D sample showed higher β content than the 25P6A4D sample, since the 25P6A4D sample had a higher viscosity than the 25P7A3D and 25P5A5D samples. This means that the β content in the PG PVDF fibers was a result of the combined effect of solution viscosity, surface tension and polarity.

### 3.3. Water Repellency

**Figure 8:**
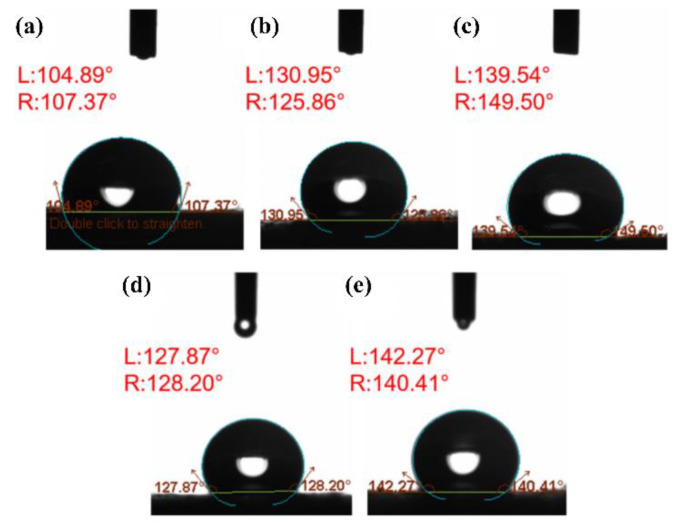
Water contact angle of: (**a**) commercial face masks; (**b**) 25P7A3D; (**c**) 20P7A3D; (**d**) 25P6A4D; (**e**) 25P5A5D.

**Figure 9:**
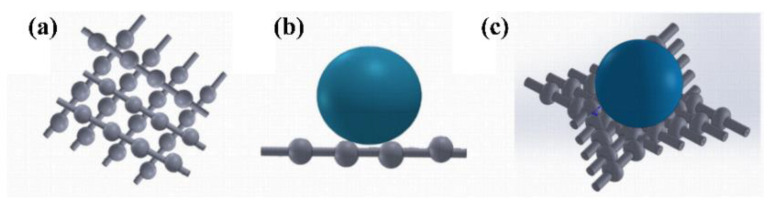
(**a**) Model of PVDF beads fiber mat; (**b**) the lateral view of the water droplet on PVDF beaded fiber mat (Cassie and Baxter model) [32]; (**c**) 3D model of the water droplet on PVDF beads fiber mat.

Theoretically, the wetting properties of a material are determined by the chemical composition and the roughness of the surface [31]. Figure 8 shows that the water contact angles of PG PVDF fiber mats (all above 125°) were much larger than that of commercial face masks (approximately 107°). To illustrate the underlying reasons, the Cassie and Baxter model is useful in this research, where the wettability of hydrophobic materials will decrease with increasing surface roughness [32]. Firstly, PVDF is a hydrophobic material, and fluorocarbons show high resistance to interact with both polar and non-polar molecules, owing to the low polarizability of the fluorine–carbon bond [33]. Secondly, the PG PVDF fiber mats have intrinsic surface roughness. The beads formed on the fibers during the PG process can trap air between the fiber mats surface and the water droplet, preventing the water from penetrating into the fiber mat (Figure 9). For these two reasons, the PG PVDF fiber mats in this work have much higher water contact angles than the outer layer of the commercial face masks, and this indicates its excellent fluid barrier properties.

As shown in Figure 8b–e, the hydrophobicity of the 20P7A3D sample (WCA ≈ 149°) was superior to that of the 25P7A3D sample (WCA ≈ 126°), while 25P6A4D had smaller water contact angles than the 25P7A3D and 25P5A5D samples. As shown in SEM results, bead density increased with decreasing polymer concentrations, and the 25P6A4D sample had the lowest bead density. Therefore, the Cassie and Baxter model can be applied in this research where higher surface roughness was caused by increased bead density, thus contributing to better hydrophobicity [32]. Consequently, by changing solution parameters, the hydrophobicity of PG PVDF fibers can be favorably adjusted to meet the fluid barrier need of face masks.

### 3.4. Yields and Production Rate

**Figure 10:**
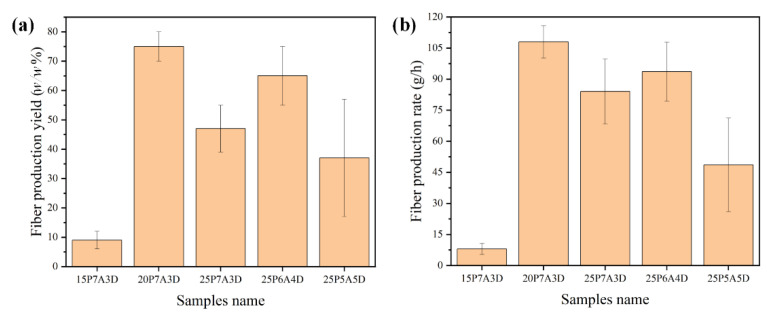
(**a**) Fiber production yield; (**b**) fiber production rate of solutions used in this work.

As shown in Figure 10a,b, the fiber production yield and the fiber production rate showed a substantial difference among groups with different solution parameters. The fiber production yield of the 15P7A3D sample was only approximately 10%, since less chain entanglement at this polymer concentration caused fewer fibers to be formed. Nonetheless, the fiber production rate of most of the samples (20P7A3D, 25P7A3D, 25P6A4D) were much higher than that of fibers produced by centrifugal spinning (0.06 kg/h) [9]. Additionally, it was reported that the production rate of PG can reach to 6 kg/h [9]. Even though the production rate of solution blowing is larger, at 7–8 kg/h, larger PG vessels with more orifices and higher volumes can be designed. Thus, the fiber production rate can be increased exponentially, while there are only one fiber emerging from a single needle in blow spinning. The fiber production yield and the fiber production rate of the 25P6A4D sample were also higher than that of fibers produced by other solvent ratios. Apart from the influence of viscosity and solvent evaporation during the fiber formation process, the larger fiber diameter of the 25P6A4D sample made it easier to be collected. Thus, there was larger fiber production yield. The 20P7A3D sample showed the largest fiber production yield and fiber production rate, which indicated that a higher solvent evaporation rate would be conducive to fiber formation. The relatively large difference in production yield of PG PVDF can be observed (see standard deviations in Figure 10), especially for the 25P5A5D sample, which may result from the instability of the polymer flow due to the introduction of airflow. Nevertheless, this problem can be tackled by using a PG setup with nozzles [27].

## 4. Conclusions

In this research, bead-on-string PVDF fibers were successfully produced by PG, and the influence of solution parameters (e.g., polymer concentrations and solvent ratio) on the fiber morphology and crystalline phases were investigated. Higher bead density and thinner fiber diameter (2.3 μm) can be obtained by decreasing the polymer concentration to 15% *w*/*v*. By changing the solvent ratio, the spinning solution with different viscosity values can be obtained, thus forming fibers with variable diameters and bead densities. All of the beaded fiber samples presented surface roughness and surface nanopores, which is beneficial for increasing the contact area between particle matters and the fiber filter. The bead sizes ranged from 60.9 μm to 88.5 μm, which indicated that it may improve the breathability by separating the closely packed fibers. The β-phase contents increased with increasing DMF concentrations, reaching 75.42% for the 25P5A5D samples. This indicated the potential for PG to produce PVDF masks with self-charging properties. The water contact angles of PVDF fibers, especially 20P7A3D at 149.5°, was higher than that of commercial face masks (approximately 107°). This shows the excellent water repellency of the PVDF fibers, which can prevent the adherence of aerosol on the PG PVDF masks. Even though the processing parameters for PG PVDF fibers still need to be optimized to improve the fraction of the β-phase, it is doubtless that this work validated the feasibility and benefit of PG PVDF fibers in reusable masks, owing to its excellent bead-on-string fibers structure and hydrophobicity. In future work, by optimizing PG processing parameters, the optimal trade-off of fiber properties, including thinner fibers, higher bead densities, higher β-phase concentration, and better mechanical properties can be obtained. The resulting β-phase content and hydrophobicity of PG PVDF fibers can offer potential for better electrostatic filtration efficiency and fluid barrier properties when compared to conventional commercially available face masks. At the same time, the bead-on-string structure showed potential for improved mechanical filtration efficiency.

## Figures and Tables

**Table 1:** Details of prepared solutions.

Sample Name	Polymer Concentrations [% (*w*/*v*)]	Acetone to DMF Ratio [*v*/*v*]	Viscosity [mPa s]	Shear rate [s^−1^]
15P5A5D	15	5/5	17.80 ± 0.21	34
15P6A4D	15	6/4	19.74 ± 0.25	34
15P7A3D	15	7/3	17.33 ± 0.20	34
20P5A5D	20	5/5	30.70 ± 0.67	12
20P6A4D	20	6/4	55.29 ± 3.80	12
20P7A3D	20	7/3	45.43 ± 3.56	12
25P5A5D	25	5/5	100.51 ± 1.95	6
25P6A4D	25	6/4	140.80 ± 17.01	5
25P7A3D	25	7/3	102.00 ± 1.39	6

## Data Availability

The authors declare data availability.
